# Implementation of an AI-Assisted Workflow for Rehabilitation Discharge Summary Creation: Evaluation of Documentation Efficiency, Usability, and Hallucinations in a 290-Bed General Hospital in Japan

**DOI:** 10.7759/cureus.109247

**Published:** 2026-05-19

**Authors:** Masaji Nakano, Takuto Imura

**Affiliations:** 1 Rehabilitation, Soseikai General Hospital, Kyoto, JPN

**Keywords:** ai hallucination, clinical documentation improvement, generative artificial intelligence, physical medicine & rehabilitation, workflow efficiency

## Abstract

Background

The widespread adoption of electronic health records (EHRs) has imposed substantial documentation burdens on healthcare workers and is internationally recognized as a major contributor to clinician burnout. Rehabilitation therapists also experience documentation burdens comparable to those of physicians and nurses. However, integrated evaluations of the efficiency, usability, and safety of generative artificial intelligence (AI)-assisted creation of rehabilitation discharge summaries remain limited.

Objective

This study aimed to evaluate the impact of an AI-assisted rehabilitation discharge summary creation workflow - combining a commercial generative AI service with an in-house Excel/Visual Basic for Applications (VBA) macro-based transcription system (Microsoft Corp., Redmond, Washington) - on documentation efficiency, usability, and safety in the rehabilitation department of a general hospital in Japan.

Methods

This single-center, before-after implementation study was conducted in the rehabilitation department of a 290-bed general hospital in Kyoto, Japan. Documentation time for rehabilitation discharge summary creation (pre-implementation, n=16; post-implementation, n=22; therapists, n=21) was analyzed using a linear mixed-effects model with random intercepts for therapist ID, supplemented by the Wilcoxon signed-rank test. Usability was assessed using the System Usability Scale (SUS) administered to 18 cooperating therapists (physical, occupational, and speech-language therapists) following implementation, with results interpreted according to the established criteria. Hallucination and omission evaluation was performed on 11 randomly sampled AI-generated rehabilitation discharge summaries by two independent raters using a published clinical safety framework; disagreements were resolved through consensus discussion.

Results

The median documentation time decreased from 23.0 minutes pre-implementation to 10.0 minutes post-implementation. The linear mixed-effects model revealed a significant reduction of 12.26 minutes (95% CI: -15.71 to -8.81; p<0.001). The Wilcoxon signed-rank test in the nine therapists who contributed to both periods also confirmed a large effect size (r=0.87, p=0.014). No difference in documentation time was observed based on previous lecture attendance (β=0.05, p=0.98). The median SUS score was 83.75 (IQR: 72.5-90.0), corresponding to Letter Grade B (80-89), with 15 of 18 respondents (83.3%) classified as "Acceptable" (≥70). In the hallucination and omission evaluation, 9 of 11 rehabilitation discharge summaries (81.8%) contained at least one error, yielding a total of 19 hallucinations and 1 omission. Major errors accounted for 52.6% (10/19), and the major rate of fabrication-type hallucinations was 83.3% (5/6).

Conclusions

This exploratory, single-center implementation study found that an AI-assisted rehabilitation discharge summary workflow was associated with reduced documentation time and favorable usability ratings. However, hallucinations occurred at a level requiring therapist verification and correction, with clinically relevant errors in high-risk content segments. These findings provide preliminary evidence that a low-cost, low-technical-barrier AI-assisted documentation workflow may improve documentation efficiency and usability within the studied institutional context. Safe implementation requires final human verification and risk-stratified review of high-risk content segments.

## Introduction

The widespread adoption of electronic health records (EHRs) has improved the quality and safety of medical care; however, it has simultaneously imposed substantial documentation burdens on healthcare providers and is internationally recognized as a major contributor to clinician burnout. A recent scoping review of 153 sources by Levy et al. conceptualized documentation burden as a challenge affecting all health professionals and defined excessive documentation burden as stress and unnecessarily heavy workload arising from misalignment between documentation systems, documentation activities, and care delivery [1]. The review further identified six contributory domains - usability, quality, regulatory, reimbursement, self-imposed, and interoperability/standards - with usability positioned as a cross-cutting factor underlying all other domains [1]. A time-and-motion study examining time allocation in ambulatory practice observed 57 physicians across four specialties for 430 hours and reported that 49.2% of clinical time was spent on EHR-related tasks and desk work, while only 27.0% was devoted to direct face-to-face patient care [2]. Furthermore, physicians spent an additional one to two hours each evening on EHR-related work after returning home, indicating that the documentation burden extends beyond working hours into personal time. In a systematic review focused on primary care, Budd identified five major contributors to EHR-related burnout (time demands, documentation and clerical burdens, complex usability, cognitive load, and electronic messaging volume) [3]. Among these, documentation has been positioned as the most prominent driver of EHR-related burnout, representing a shared challenge across healthcare settings worldwide.

Compared to research focused on physicians, studies on rehabilitation therapists (physical, occupational, and speech-language therapists) remain limited. Nevertheless, therapists also experience EHR documentation burdens comparable to those of physicians and nurses. In a qualitative descriptive study and quality improvement initiative conducted at an orthopedic specialty hospital in the United States, Schwartz-Dillard et al. reported that outpatient rehabilitation therapists experienced documentation burdens similar to those documented for physicians and nurses, and that manual data entry imposed a burden on therapists' time and clinical care [4]. Rehabilitation discharge summaries are documents that require structured information across multiple segments, including the patient's clinical course, functional assessments, activities of daily living, and rehabilitation plans, and their preparation requires considerable time. In Japan, although rehabilitation therapists are presumed to face similar documentation burdens, quantitative evidence in this context has not been sufficiently accumulated.

The rapid advancement of large language models (LLMs) in recent years has prompted growing application attempts in clinical, educational, and research contexts within the medical field. In a review of LLM applications in medicine, including ChatGPT, Thirunavukarasu et al. summarized the strengths, limitations, and current state of clinical applications [5]. Among potential applications, digital scribe systems, which use LLMs to automatically generate clinical documents, have attracted attention as a promising approach to alleviating documentation burdens. van Buchem et al. evaluated a digital scribe system (Autoscriber) developed in the Netherlands through summary creation tasks performed by 22 medical students with clinical experience using simulated consultations [6]. Although automated summarization with manual editing demonstrated potential for reducing documentation time without compromising quality compared to manual summarization, considerable individual variability was observed, and not all users benefited from time savings. Recent evidence from allied health private practice suggests that AI scribes may reduce documentation burden and improve clinician-patient interactions, indicating that AI-assisted documentation may be relevant beyond physician-centered settings [7]. In Japanese rehabilitation settings, a pilot study has begun to explore the impact of generative AI on clinical documentation, although evidence remains preliminary [8]. However, generative AI carries critical clinical safety concerns, including hallucinations (generation of factually unfounded information) and information omissions, particularly in medical document applications, where quantitative evaluation of these risks and the design of human verification processes are indispensable. Nevertheless, integrated evaluations of the efficiency, usability, and safety of generative AI-assisted rehabilitation discharge summary creation, specifically focusing on the rehabilitation field, remain limited.

This study was conducted in the rehabilitation department of a 290-bed general hospital in Kyoto, Japan, and was designed as an exploratory, single-center implementation study. Rather than evaluating the isolated performance of a specific generative AI model, this study aimed to evaluate the feasibility, efficiency, usability, and safety profile of an AI-assisted rehabilitation discharge summary creation workflow implemented in routine clinical practice. The workflow combined a hospital-available generative AI service (Ubie, Tokyo, Japan) with an in-house developed Excel/Visual Basic for Applications (VBA) macro-based transcription system (Microsoft Corp., Redmond, Washington, USA). Specifically, three primary outcomes were defined: (1) reduction in documentation time, assessed using a before-after comparison with a linear mixed-effects model, (2) usability, assessed using the System Usability Scale (SUS), and (3) occurrence of hallucinations and omissions, assessed using the framework of Asgari et al. [9]. The conceptual contribution of this study lies not in geographic novelty alone, but in evaluating a low-cost, low-technical-barrier integration architecture for AI-assisted clinical documentation in a resource-constrained rehabilitation environment.

## Materials and methods

Study design and setting

This exploratory, single-center, before-after implementation study was conducted in the rehabilitation department of a 290-bed general hospital in Kyoto, Japan. In Japan's medical reimbursement system, disease-specific rehabilitation fees are categorized into five classifications: cardiovascular disease, cerebrovascular disease and others (CVA), disuse syndrome (DUS), musculoskeletal disorders (MSK), and respiratory disease. At our institution, three of these classifications - CVA, MSK, and DUS - are billed, and these three categories account for approximately 90% of all rehabilitation cases. For these regulatory and operational reasons, all subjects in this study fell into one of these three categories. This study was approved by the Institutional Review Board of Soseikai General Hospital (approval number: Rin-R 8-09). All participants provided informed consent for the research use of their data.

AI-assisted summary creation workflow

At our institution, draft rehabilitation discharge summaries were generated in text format from clinical information using Ubie generative AI, a hospital-available healthcare generative AI service (Ubie, Tokyo, Japan). Because Ubie generative AI is a vendor-managed service, the underlying language model version, inference parameters, token-level processing settings, and proprietary internal architecture were not available to the investigators. Therefore, the present study evaluated the implemented clinical workflow as used in routine practice, rather than the isolated technical performance of a specific language model. The specific integration method between this service and the hospital's electronic medical record (EMR) system was outside the scope of this study. We evaluated the entire workflow from when therapists obtained the final text output to the finalization of rehabilitation discharge summaries by transcription into templates.

The output of Ubie was text-only and could not be directly inserted into the standardized rehabilitation discharge summary templates used in routine clinical practice. To bridge this gap, we developed an in-house Excel/VBA macro-based system (version 8.3) that automatically transcribes AI-generated text into the templates with a single click.

The prompt instruction (version 4.0) provided to the generative AI was designed in-house and consisted of structured information extraction across nine segments: disease name, date of onset/injury, comorbidities, past medical history, physical function, cognition, swallowing and language, ADL precautions, and clinical course. The prompt was designed to organize EMR-derived clinical information into these predefined segments and to output text compatible with the institutional rehabilitation discharge summary workflow. The full prompt instruction used in this study, including the original Japanese version and an English translation, is provided in Appendices. Because many AI-generated rehabilitation discharge summaries may contain hallucinations, the workflow was operated such that all AI-generated rehabilitation discharge summaries were verified and corrected by the responsible therapist before finalization. For items such as comorbidities and past medical history, the AI-generated output presented extracted candidate items, from which the responsible therapist selected clinically appropriate information rather than accepting them automatically. The post-implementation documentation time included this verification, correction, selection of candidate items, and finalization process, rather than only the time required to generate or transcribe the AI output.

Implementation process

For the introduction of the AI-assisted workflow, a PDF-format operating manual describing the system usage was distributed to all staff members. Subsequently, individual usage lectures were provided to therapists who requested them, and the researchers recorded which therapists had received the lecture. As a result, during post-implementation operations, therapists who had received the lecture and those who had not coexisted in the workflow, and the lecture status of each therapist at the time of rehabilitation discharge summary creation, could be linked to the data.

Participants and data collection

Documentation time data were collected from physical therapists (PTs), occupational therapists (OTs), and speech-language therapists (STs) working in the rehabilitation department. Each therapist was asked to self-measure the time required from the start of rehabilitation discharge summary creation to its completion, including corrections, using a stopwatch. Because documentation time was self-measured in routine clinical practice, recorded times may have varied according to individual timing practices, interpretation of the start and end points, and increased measurement awareness, particularly during the early implementation period.

The number of rehabilitation discharge summaries created was 16 in the pre-implementation period and 22 in the post-implementation period. Because multiple rehabilitation discharge summaries are typically created by the same therapist, an individual therapist could contribute multiple rehabilitation discharge summaries to either or both periods. To appropriately handle this structure, an anonymized therapist ID was assigned to each rehabilitation discharge summary. Each rehabilitation discharge summary was classified by disease category (CVA/MSK/DUS).

For post-implementation cases, whether the responsible therapist had received the optional in-house lecture before rehabilitation discharge summary creation was additionally recorded.

Outcome measures

*Primary Outcome: Documentation Time* The primary outcome was the time required to create one rehabilitation discharge summary (in minutes). Documentation time was selected as the primary efficiency outcome because, in a recent systematic review of 135 studies on documentation burden measurement by Murad et al., time-related measures, including overall time spent in the electronic health record, time on clinical documentation activities, and after-hours work, were identified as the most frequently used quantitative indicators of documentation burden [10]. As described above, the therapists self-measured the time from the start of rehabilitation discharge summary creation to its completion using a stopwatch.

System Usability Scale (SUS)

The SUS [11] was distributed as an anonymous paper questionnaire to all therapists following implementation. To ensure anonymity, therapists submitted completed questionnaires anonymously into a collection box, which was opened only after data collection was completed. Demographic information, such as years of experience and age, was not collected to ensure anonymity. Ultimately, responses were obtained from 18 cooperating therapists (PTs, OTs, and STs), which does not represent responses from all therapists in the post-implementation period. All 18 respondents completed the SUS immediately after their first use of the system. SUS scores were calculated using the standard method (range: 0-100) and interpreted based on the three evaluation criteria (adjective rating scale, acceptability range, and school grading scale) presented in Figure 4 of Bangor et al. [12]. The acceptability range was defined as acceptable (≥70), marginal (50-69), and not acceptable (<50).

Hallucination and omission evaluation

Eleven AI-generated rehabilitation discharge summaries (selected randomly to ensure distribution across the three disease categories) were evaluated for reference-based hallucination and omission using the clinical safety evaluation framework proposed by Asgari et al. [9]. This evaluation aimed to confirm the presence of hallucinations as a minimum safety indicator among the multiple methods of evaluating the completeness of AI-generated rehabilitation discharge summaries. Each rehabilitation discharge summary was independently evaluated by two physical therapists (M.N. and T.I.), who compared each statement in the AI output against the original medical records. Errors were classified into hallucinations and omissions according to the error classification of Asgari et al. [9]. Hallucinations were further categorized into four types (fabrication, negation, contextual, and causality), and omissions were categorized into three types (current issues, PMFS (past medical history, medications, family history, and social background), and information and plan). Each error was classified as major (affecting diagnosis or management) or minor (without direct impact) based on the basic classification of Asgari et al. [9]. Although the original Asgari study also employed a five-tier consequence classification (catastrophic/major/considerable/significant/minor), our study targeted rehabilitation discharge summaries, where situations directly leading to catastrophic outcomes (death or permanent disability) in the five-tier classification are unlikely. Therefore, only the basic two-tier classification (major/minor) was adopted. The operational criterion for major classification was defined as "whether the relevant statement, if used unmodified in the rehabilitation discharge summary, could cause problems in patient management (rehabilitation planning, risk management, handover, etc.)." In the previous study by Asgari et al. [9], inter-rater disagreements were resolved through third-party adjudication by senior clinicians with more than 20 years of clinical experience. In our study, due to resource constraints, the two raters discussed disagreement items and reached consensus to finalize judgments.

Statistical analysis

Documentation Time Analysis

Because the same therapist created multiple rehabilitation discharge summaries, observations were not independent. To appropriately handle this clustering structure, a linear mixed-effects model (LMM) was used as the primary analysis. The fixed effect was the implementation period (pre vs. post), and a random intercept was set for therapist ID. Sensitivity analyses were performed by sequentially adding disease category, profession, and years of experience as covariates to assess robustness. As a supplementary analysis, the average documentation time per therapist was compared using the Wilcoxon signed-rank test, restricted to therapists who created rehabilitation discharge summaries in both periods, and the matched-pair effect size r was calculated.

Subgroup Analysis

Among post-implementation cases, the documentation time of rehabilitation discharge summaries created by therapists who had received the lecture and those who had not was compared using an LMM adjusted for disease category as a covariate.

SUS and Hallucination/Omission Evaluation

SUS scores and hallucination/omission results were reported using descriptive statistics with median and interquartile range (IQR).

General Considerations

Normality was confirmed using the Shapiro-Wilk test. All analyses were performed using R (version 4.5.3, R Foundation for Statistical Computing, Vienna, Austria; https://www.R-project.org/) [13] in an RStudio environment. Two-sided p < 0.05 was considered statistically significant.

## Results

Documentation time

The analysis included 38 rehabilitation discharge summaries (16 pre-implementation and 22 post-implementation) created by 21 therapists (PT=17, OT=1, ST=3). Of these, nine therapists contributed rehabilitation discharge summaries to both pre- and post-implementation periods, three contributed only pre-implementation, and nine contributed only post-implementation.

**Figure 1:**
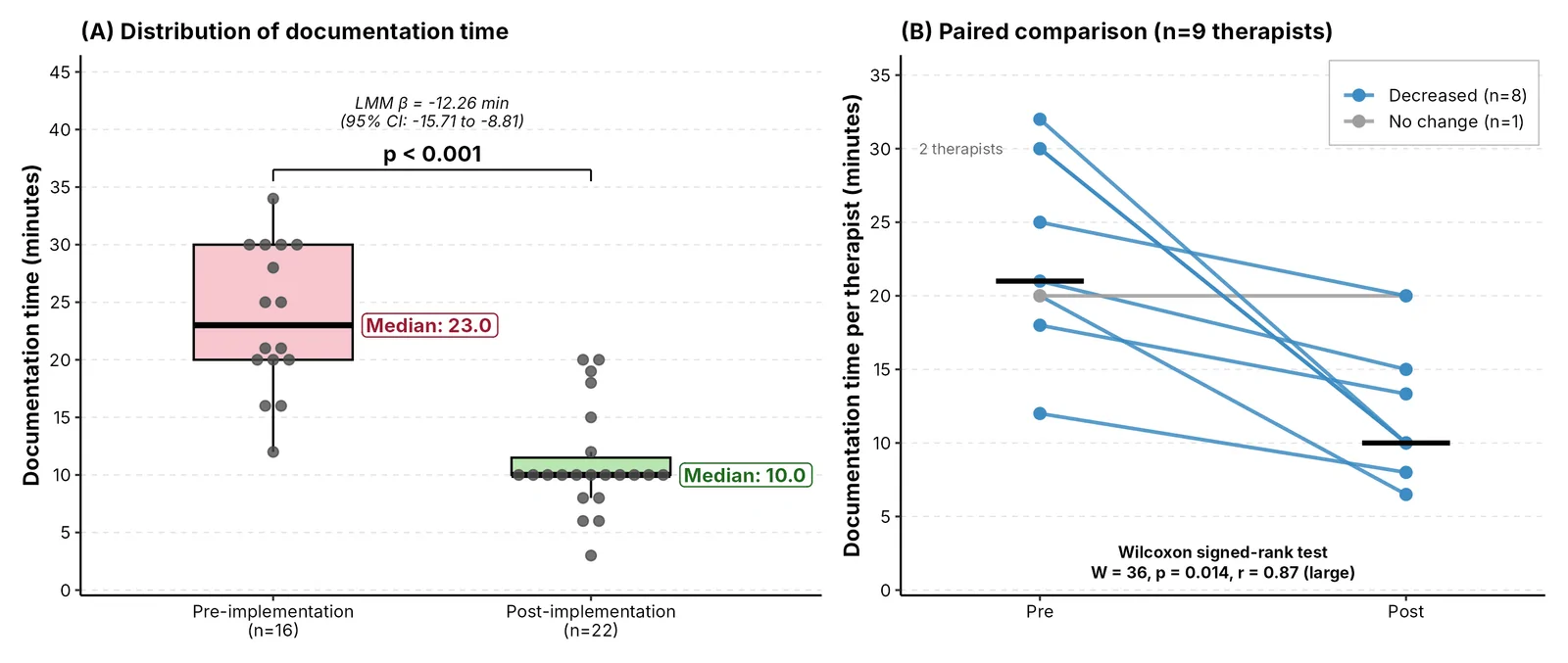
Documentation time for rehabilitation discharge summaries before and after AI-assisted workflow implementation (A) Distribution of documentation time pre-implementation (n=16) and post-implementation (n=22). Box plots show median (line within box), interquartile range (box), and 1.5× IQR (whiskers). Individual data points are overlaid as dots. The linear mixed-effects model adjusted for therapist clustering revealed a reduction of 12.26 minutes (95% CI: -15.71 to -8.81; p<0.001). (B) Paired comparison among nine therapists who contributed to both periods. Eight showed reduced documentation time (blue), one showed no change (gray), and none showed an increase (Wilcoxon signed-rank test: W=36, p=0.014, r=0.87, large effect size). Horizontal black bars indicate the medians of the therapists’ mean documentation times. Two therapists had identical values (30.0 to 10.0 minutes), and their lines overlap. LMM: linear mixed-effects model; CI: confidence interval; IQR: interquartile range

The median documentation time decreased from 23.0 minutes (IQR: 20.0-30.0) pre-implementation to 10.0 minutes (IQR: 10.0-11.5) post-implementation (Figure 1A). The primary analysis using LMM (random intercepts for therapist ID) revealed a significant reduction of 12.26 minutes in post-implementation documentation time (95% CI: -15.71 to -8.81; p<0.001). The sensitivity model adjusted for disease category, profession, and years of experience showed essentially the same effect size (adjusted β = -11.80 minutes; 95% CI: -15.19 to -8.40; p<0.001).

As a supplementary analysis, the Wilcoxon signed-rank test restricted to the nine therapists who contributed to both periods also confirmed a significant reduction (W=36, p=0.014), with a large matched-pair effect size (r=0.87). Among the nine paired therapists, eight showed reduced documentation time, one showed no change, and none showed an increase (Figure 1B).

Table 1 summarizes the comparison of documentation time before and after implementation.

**Table 1:** Comparison of rehabilitation discharge summary documentation time before and after AI-assisted workflow implementation IQR: interquartile range; LMM: linear mixed-effects model; CI: confidence interval

	Pre (n=16)	Post (n=22)	LMM β (95% CI)	p
Documentation time (min), median (IQR)	23.0 (20.0-30.0)	10.0 (10.0-11.5)	-12.26 (-15.71 to -8.81)	<0.001
Wilcoxon signed-rank test (paired, n=9)	-	-	r=0.87	0.014

Lecture effect (subgroup analysis)

Of the 22 post-implementation cases, 16 rehabilitation discharge summaries were created by therapists who had not received the lecture, and 6 were created by therapists who had received the lecture. The median documentation time was nearly identical between the two groups (no lecture: 10.0 min, IQR: 9.5-12.75; with lecture: 10.0 min, IQR: 10.0-10.0). The LMM adjusted for disease category showed no between-group difference (β=0.05 min, 95% CI: -4.49 to 4.59; p=0.98). This result suggests that no clear difference in documentation time was observed according to previous lecture attendance.

System Usability Scale (SUS)

The SUS was completed by 18 cooperating therapists (PTs, OTs, and STs) immediately after their first use of the AI-assisted workflow. This represented the number of respondents to the anonymous questionnaire distributed to all post-implementation therapists. The median SUS score was 83.75 points (IQR: 72.5-90.0; range: 57.5-100.0; mean 81.81, SD = 12.12). According to the evaluation criteria of Bangor et al. [12], this median score corresponds to Letter Grade B (80-89) and approaches the "excellent" rating (mean: 85.5 points) on the Adjective Rating Scale. In terms of acceptability, 15 of 18 respondents (83.3%) were classified as "acceptable" (≥70 points), while three were classified as "marginal" (50-69 points), and no respondents were classified as "not acceptable" (<50 points).

Hallucination and omission evaluation

**Figure 2:**
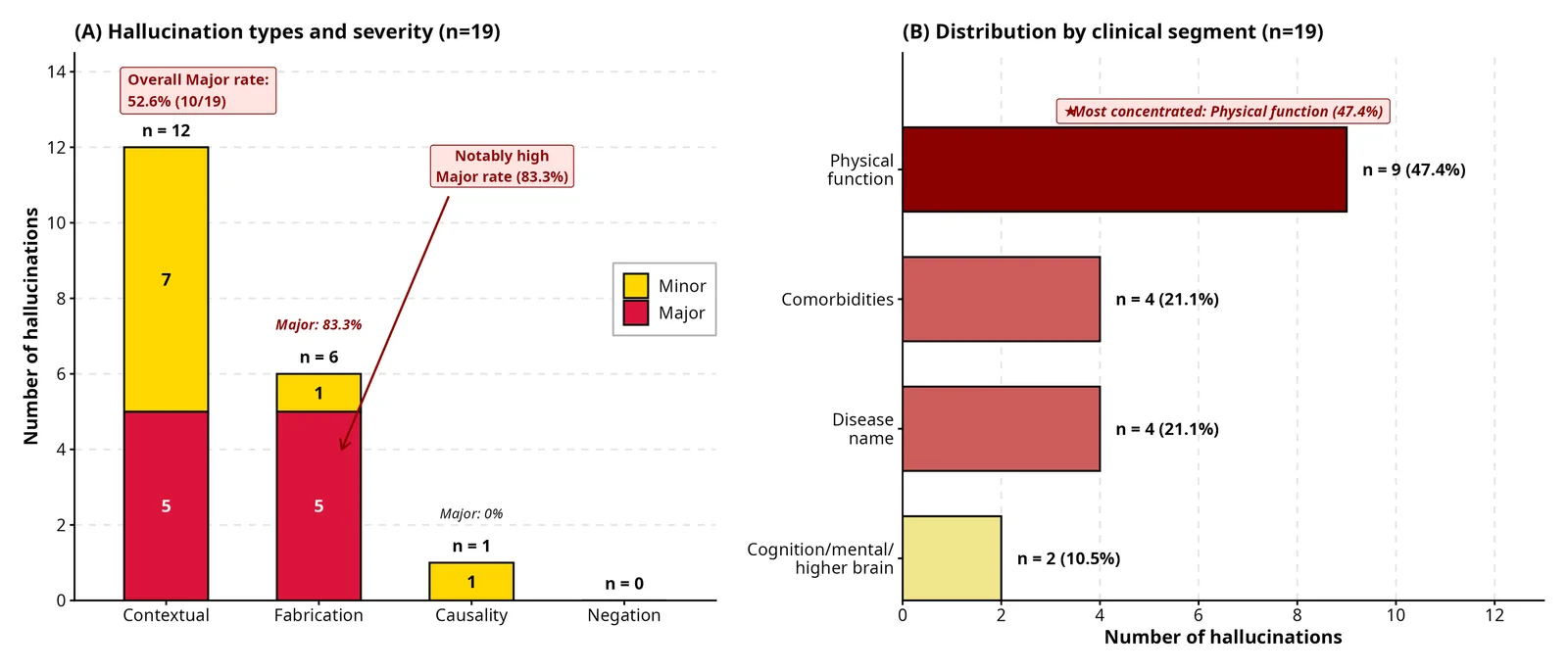
Hallucination types, severity, and clinical segment distribution (A) Distribution of 19 hallucinations across four error types (contextual, fabrication, causality, negation), with severity classification (major in red, minor in yellow). The overall major rate was 52.6% (10/19); fabrication-type hallucinations showed a notably high major rate of 83.3% (5/6). (B) Distribution of 19 hallucinations across clinical content segments. The physical function segment was most affected (n=9, 47.4%), followed by comorbidities (n=4, 21.1%), disease name (n=4, 21.1%), and cognition/mental/higher brain function (n=2, 10.5%).

A reference-based hallucination and omission evaluation was performed on 11 randomly selected AI-generated rehabilitation discharge summaries based on the framework of Asgari et al. [9]. In the independent evaluation phase, the two raters identified a total of 24 potential errors. Through consensus discussion of disagreements, 20 errors were confirmed as true errors, while 4 (16.7%) were rejected as non-errors. Of the 11 evaluated rehabilitation discharge summaries, 9 (81.8%) contained at least one error, and 2 (18.2%) were error-free. A total of 19 hallucinations and one omission were identified. The median number of errors per rehabilitation discharge summary was 2.0 (IQR: 1.0-2.0; range: 0-5). Of the 19 hallucinations, the majority (12, 63.2%) were of the contextual type. Fabrication accounted for six (31.6%), causality for one (5.3%), and no negation type was observed. The overall major rate was 52.6%, with a notably high major rate observed in fabrication-type hallucinations (83.3%) (Figure 2A). Hallucinations were most frequently observed in the physical function segment (9/19, 47.4%), followed by comorbidities (4/19, 21.1%), disease name (4/19, 21.1%), and cognition/mental/higher brain function (2/19, 10.5%) (Figure 2B). The distribution of hallucination types and severity classifications is summarized in Table 2.

**Table 2:** Hallucination types and severity classification Severity classification was based on the basic two-tier criteria of Asgari et al. (major: affecting diagnosis or management, minor: without direct impact). Major rate (%) is calculated as major/(major + minor) within each hallucination type.

Hallucination type	n (%)	Major	Minor	Major rate (%)
Contextual	12 (63.2)	5	7	41.7
Fabrication	6 (31.6)	5	1	83.3
Causality	1 (5.3)	0	1	0
Negation	0 (0.0)	0	0	-
Total	19 (100)	10	9	52.6

The single omission occurred in a cerebrovascular disease case in the comorbidities segment, classified as a major error in the PMFS (past medical history, medications, family history, and social background) category in the Asgari et al.'s classification [9]. The distribution of hallucinations by clinical content segment is shown in Table 3.

**Table 3:** Distribution of hallucinations by clinical segment n=19 hallucinations across 11 evaluated AI-generated rehabilitation discharge summaries. Percentages are calculated as n/19.

Clinical segment	n	% (overall)
Physical function	9	47.4
Comorbidities	4	21.1
Disease name	4	21.1
Cognition/mental/higher brain function	2	10.5
Total	19	100

By disease category, the mean number of errors per case showed an increasing trend in the order of MSK (six cases, one per case), CVA (three cases, two per case), and DUS (two cases, four per case). Both error-free cases were musculoskeletal disorders. The distribution of errors by disease category is presented in Table 4.

**Table 4:** Distribution of errors by disease category Mean errors per case are calculated as hallucinations + omissions/n. Note: DUS comprised only two cases, and interpretation of the mean errors per case requires caution. CVA: cerebrovascular disease and others; MSK: musculoskeletal disorders; DUS: disuse syndrome

Disease category	n	Error-free	Hallucinations	Omissions	Mean errors
Cerebrovascular (CVA)	3	0	5	1	2.00
Musculoskeletal (MSK)	6	2	6	0	1.00
Disuse syndrome (DUS)	2	0	8	0	4.00

## Discussion

This exploratory, single-center implementation study evaluated an AI-assisted rehabilitation discharge summary creation workflow that combined Ubie generative AI, a hospital-available healthcare generative AI service, with an in-house developed Excel/VBA macro-based transcription system in the rehabilitation department of a 290-bed general hospital in Japan. The study examined three dimensions: efficiency, usability, and safety. The workflow was associated with a significant reduction in documentation time (LMM β = -12.26 minutes, 95% CI: -15.71 to -8.81, p<0.001) (Figure 1), a large effect size in the matched-pair analysis (Wilcoxon r=0.87), and favorable usability ratings (median SUS: 83.75, corresponding to Letter Grade B and approaching the "excellent" adjective rating). Concurrently, the hallucination evaluation revealed that approximately half of the identified hallucinations were classified as major (52.6%), with a notably high major rate observed in fabrication-type hallucinations (83.3%) (Figure 2). These findings suggest that, within the studied institutional context, the AI-assisted workflow may improve documentation efficiency and usability; however, the safety profile of AI-generated draft content requires careful management.

The substantial reduction in documentation time observed in our study is consistent with previous reports of AI scribe technologies in medical settings, although direct comparisons must account for differences in clinical context. Shah et al. [14] reported a three-month pilot study at Stanford Health Care evaluating an ambient AI scribe (DAX Copilot) among 48 physicians (paired survey n=38), demonstrating significant reductions in task load (-24.42, p<0.001) and burnout (-1.94, p<0.001), as well as moderate improvement in usability (+10.9, p<0.001). In physician-centered ambulatory settings, ambient AI scribe implementation has been associated with modest but significant reductions in documentation time and total EHR time, with notable individual-level heterogeneity in benefit [15]. A prospective time-motion study in a Singaporean outpatient setting reported that ambient AI scribe use significantly reduced documentation time by 15.0% and increased clinician-patient eye contact, while overall visit cycle time did not significantly change [16]. Although the clinical context and workflow differ, these findings suggest that AI-assisted documentation can reduce documentation-related time in real-world practice. In the present study, the observed reduction in documentation time was achieved within a low-cost, low-technical-barrier configuration combining an existing hospital-available generative AI service with a lightweight, in-house developed VBA macro. This configuration may represent a practical integration approach for rehabilitation departments with limited technical resources. However, implementation feasibility may vary across institutions depending on the EMR structure, availability of extractable text data, institutional security policies, and the level of local digital or technical support. Institutions with less interoperable EMR systems or limited technical support may require additional preprocessing, manual data extraction, template customization, or maintenance resources before adopting a similar workflow. Therefore, the present findings should be interpreted as context-dependent, and future studies should evaluate implementation requirements across diverse EMR environments and resource settings.

Schwartz-Dillard et al. [4] reported that outpatient rehabilitation therapists in the United States experienced documentation burdens comparable to those of physicians and nurses, with manual data entry imposing a burden on therapists' time and clinical care. Our findings provide an early quantitative implementation-based evaluation of this internationally recognized challenge in rehabilitation. Furthermore, van Buchem et al. [6] evaluated a digital scribe system through summary creation tasks performed by 22 medical students using simulated consultations and reported considerable individual variability in time savings, with not all users benefiting from time reductions. Of particular relevance, Omon et al. recently conducted a before-after pilot study in two Japanese rehabilitation hospitals using a generative AI tool for SOAP-format clinical documentation (n=12 therapists), and reported that AI-assisted documentation significantly reduced perceived time pressure (NASA-TLX) and improved documentation quality, although overall documentation time did not differ significantly between conditions (305 s vs. 336 s, n.s.) [8]. The authors attributed the absence of time savings to insufficient voice-input proficiency among users and the time required for editing AI-generated drafts. In contrast, our workflow was associated with a significant reduction in actual rehabilitation discharge summary creation time, which may reflect differences in document type (rehabilitation discharge summaries requiring longitudinal clinical information integration vs. SOAP-format clinical documentation), workflow design, and the use of a macro-based transcription system that minimized manual transcription into institutional templates.

Although the AI-assisted workflow was associated with improvements in efficiency and favorable usability ratings, our hallucination evaluation revealed clinically meaningful safety concerns. Contextual hallucinations were the most frequent (12 of 19, 63.2%) (Figure 2A), characterized in our context by the persistence of outdated information (e.g., earlier or interim assessment values and assistance levels) being output as current data. The major rate within contextual hallucinations was 41.7%, indicating that while many were correctable surface-level inconsistencies, a substantial proportion still required attention to prevent management errors. Fabrications (6 of 19, 31.6%) demonstrated a notably high major rate of 83.3%, reflecting the generation of nonexistent comorbidities or unsupported diagnostic labels derived from symptoms. These errors, if left unmodified, could directly influence rehabilitation planning, risk management, or handover communication. Causality-type errors were rare (1 of 19) in our cohort, suggesting that the inappropriate linking of unrelated phenomena occurred infrequently in this rehabilitation discharge summary context. The clinical segment distribution showed that hallucinations were most concentrated in the physical function segment (47.4%), followed by comorbidities and disease names (each 21.1%). This pattern suggests that segments requiring quantitative information from longitudinal records (e.g., functional measurements over time) and segments depending on accurate medical history are particularly vulnerable to AI-generated errors. These findings support a verification process weighted toward segments and error types with elevated major-error risk, rather than uniform review of all content.

While the overall frequency of errors appeared high, these errors were identified in AI-generated draft summaries before finalization and should therefore be interpreted as potential safety risks within draft content rather than confirmed errors in finalized clinical documents. In the routine workflow, information such as comorbidities and past medical history was presented as AI-extracted candidate items, from which the responsible therapist selected clinically appropriate information rather than accepting them automatically. In addition, the entire AI-generated text was reviewed and corrected by the responsible therapist before finalization. Importantly, the post-implementation documentation time included therapist verification, correction, selection of candidate items, and finalization of the entire summary, rather than only the time required to generate or transcribe the AI output. Therefore, the observed time reduction was achieved within a human-in-the-loop workflow that retained the review process, rather than by omitting verification. In practical workflow terms, the clinical impact of these errors would depend on whether they remained uncorrected in high-risk segments, such as disease name, comorbidities, past medical history, or physical function, where they could influence rehabilitation planning, risk management, or handover communication. Overall, these considerations reinforce the need for final human verification and targeted review of high-risk content segments before clinical use.

Previous safety evaluations of AI-generated clinical documentation support this cautious interpretation. Biro et al. evaluated two commercial ambient digital scribe products in a simulated clinical setting and reported that 70% of generated draft notes contained errors, with omission errors being the most frequent across both products (83% and 54% of errors, respectively) [17]. Although the document genre and AI architecture differ from our study, the predominance of content-related errors in their evaluation is consistent with our broader finding that AI-generated clinical documentation remains vulnerable to content-related errors and cannot be finalized without human review.

The observed pattern of hallucinations also has implications for prompt design. The current prompt instruction (version 4.0) consisted of structured information extraction across nine segments, which evolved through iterative refinement to address various hallucination patterns. However, the increasing complexity of prompts - as additional constraints are added to suppress specific error types - may increase operational complexity and make outputs more difficult to standardize. A potentially more sustainable approach would be staged processing, in which separate, simpler prompts handle distinct sub-tasks (e.g., one prompt for extracting current functional status, another for cross-checking comorbidity lists), with intermediate validation between stages. Using a staged prompting approach, with separate prompts for specific sub-tasks such as extracting current functional status and cross-checking comorbidity lists, could plausibly reduce the major-error rate in AI-generated draft summaries and thereby reduce the cognitive burden on therapists during verification. Future work should empirically test whether staged prompting reduces fabrication-type hallucinations while maintaining the efficiency gains observed in this study.

Taken together, these findings support the need to consider a human-in-the-loop operational model for AI-assisted documentation in rehabilitation. The efficiency and usability gains observed are potentially clinically meaningful: a reduction of approximately 13 minutes per rehabilitation discharge summary, multiplied across the daily caseload of a rehabilitation department, may create additional time that could be redirected toward direct patient care. However, human verification itself may impose cognitive and operational burden, including the effort required to compare AI-generated statements with source EMR information, judge the appropriateness of candidate items, and correct hallucinations or omissions. Although the post-implementation documentation time in this study included therapist verification and correction, we did not separately quantify verification time, cognitive workload, fatigue, or adherence to the review process. Future implementations should therefore monitor not only total documentation time but also the burden and reliability of human verification, and should consider risk-stratified review procedures that focus attention on high-risk segments such as disease name, comorbidities, past medical history, and physical function.

At the same time, the safety profile observed in this study, including a major-error rate exceeding 50% among hallucinations and 83.3% of fabrications classified as major, indicates that unverified release of AI-generated rehabilitation discharge summaries cannot be recommended in current practice. Operationally, our results suggest three priorities for sustainable implementation: (1) maintaining final human verification as a non-negotiable step, (2) directing verification effort toward high-risk segments and error types identified in this study, and (3) tracking major-error rates over time as a leading indicator of safety performance. Recently, Wang et al. proposed the SCRIBE evaluation framework for ambient digital scribing tools, which integrates simulation, computational metrics, reviewer assessment, and intelligent (LLM-based) evaluation to assess factuality, completeness, robustness, and fairness in AI-generated clinical documentation [18]. Although the present study did not adopt this framework prospectively, our risk-stratified verification approach, which prioritizes human review of clinically high-risk segments, such as physical function and comorbidities, aligns with the framework's emphasis on multidimensional safety assessment. Future implementations in rehabilitation settings would benefit from incorporating such structured evaluation approaches. These priorities may be implemented even within low-cost, low-technical-barrier configurations such as the one used in this study, although further validation is needed before broader adoption across different rehabilitation settings.

Limitations

Several limitations warrant acknowledgment.

First, this is a single-center study conducted at a 290-bed general hospital in Japan, and the generalizability of findings to other settings (different hospital sizes, different EMR systems, different patient populations) requires further multicenter validation.

Second, sample sizes were modest in each component: documentation time (n=38 rehabilitation discharge summaries from 21 therapists), SUS (n=18 respondents), and hallucination evaluation (n=11 rehabilitation discharge summaries). The hallucination evaluation in particular included only two cases for the DUS category, and disease-category comparisons of error rates should be interpreted cautiously. The SUS sample of 18 also represented a subset of post-implementation therapists rather than a complete census, introducing potential response-related bias.

Third, this is a before-after implementation study without a randomized control group. Although the LMM accounted for therapist-level clustering and the sensitivity analysis adjusted for disease category, profession, and years of experience, residual confounding from secular trends or co-occurring departmental changes cannot be excluded. In addition, documentation time was self-measured by therapists using a stopwatch, which may have introduced variability in recorded times due to differences in individual timing practices, interpretation of start and end points, or increased measurement awareness during the early implementation period.

Fourth, regarding the SUS Japanese version, our study did not fully follow the cross-cultural adaptation procedures of Beaton et al. [19]. We used an existing Japanese translation of the SUS rather than performing a formal forward-backward translation and expert committee review, which limits the cross-cultural validity of the usability scores. In addition, usability was assessed immediately after the first use of the AI-assisted workflow, and the stability of SUS scores during longer-term routine use was not evaluated. User perceptions may change as therapists gain familiarity with the system, encounter diverse cases, or experience cumulative verification burden; therefore, longitudinal usability assessment is needed.

Fifth, the hallucination evaluation framework we referenced [9] was originally developed for English-language audio transcripts of primary care consultations summarized by GPT-4. In contrast, our study evaluated Japanese-language rehabilitation discharge summaries generated from EMR-derived inputs using Ubie generative AI, a vendor-managed healthcare generative AI service. Therefore, the input format, document genre, language, clinical context, and type of generative AI system differed substantially between the two settings. Direct numerical comparisons with the original Asgari values (e.g., 1.47% hallucination rate and 44% major rate) should therefore be interpreted with caution. Japanese-specific linguistic features, such as particle omission, honorific expressions, and medical abbreviations, may have influenced hallucination patterns. In addition, the integration mechanism between Ubie generative AI and the EMR system was outside the scope of evaluation in this study.

Sixth, the reproducibility of the AI component is limited by the vendor-managed nature of the generative AI service. The underlying language model version, inference settings, token constraints, and proprietary internal processing architecture were not accessible to the investigators. In addition, because the EMR integration mechanism was outside the scope of this study, the relative contributions of input formatting, AI generation, therapist verification, and Excel/VBA-based transcription could not be fully disentangled. Therefore, the findings should be interpreted as an evaluation of the implemented clinical workflow rather than the isolated technical performance of a specific generative AI model. In addition, only the final operational version (version 4.0) of the prompt instruction is reported in Appendices. Earlier iterations were refined in-house to suppress recurrent hallucination patterns and to standardize the tagged output format, but the intermediate versions were not systematically archived for formal version-by-version comparison.

Seventh, this study originally planned a two-stage evaluation comprising qualitative assessment of AI-generated rehabilitation discharge summaries (clinical utility, comprehensiveness of information, and reader-rated quality) and hallucination evaluation; however, due to time constraints during the study period, only the hallucination evaluation was completed as a minimum safety indicator. The qualitative dimensions of rehabilitation discharge summaries were not evaluated in the present study, and future multidimensional assessment is required.

Eighth, we did not formally calculate inter-rater agreement metrics such as Cohen's kappa [20] for hallucination evaluation, because the evaluation structure (in which only flagged items were reviewed) made statistically valid κ calculation difficult. Instead, following the approach used in the original Asgari study [9], we ensured evaluation reliability through transparent consensus discussion.

## Conclusions

This exploratory, single-center implementation study evaluated a rehabilitation discharge summary creation workflow that combined Ubie generative AI, a hospital-available healthcare generative AI service, with an in-house developed Excel/VBA macro-based transcription system in the rehabilitation department of a 290-bed general hospital. The workflow was associated with a significant reduction in documentation time (LMM β = -12.26 min, p<0.001; Wilcoxon r=0.87) and favorable usability ratings (median SUS: 83.75; 83.3% rated as "acceptable"). No clear difference in documentation time was observed according to previous lecture attendance. Conversely, the hallucination evaluation identified clinically relevant safety concerns: 52.6% of hallucinations were classified as major, contextual hallucinations accounted for the largest proportion, and fabrication-type hallucinations showed a high major rate (83.3%). Errors were particularly frequent in the physical function segment. These findings provide preliminary evidence that a low-cost, low-technical-barrier AI-assisted documentation workflow may improve documentation efficiency and usability in the studied institutional context, while also demonstrating that AI-generated rehabilitation summaries require final human verification. Safe implementation should therefore be based on a human-in-the-loop workflow and risk-stratified verification of high-risk content segments. Future research should assess generalizability through larger multicenter validation, further evaluate verification burden and longitudinal usability during sustained use, mitigate hallucinations through simplified or staged prompt design, and incorporate multidimensional evaluation, including qualitative aspects of rehabilitation discharge summaries.

## Appendices

Prompt instruction version 4.0 used in this study

The following prompt instruction was used in routine operation during the study period. The original Japanese prompt is shown first, followed by an English translation prepared by the authors for the reader understanding. The original operational prompt was written in Japanese.

A. *Original Japanese Prompt*

# リハビリサマリー生成プロンプト（運用版 v4.0）

あなたは電子カルテ内の記録を参照できる前提で作業すること。 目的は、医学知識のない人でも「なぜ介助が必要か」「どこが危険か」「どう介助すべきか」「どこまで回復したか」が分かるリハビリサマリーを作成すること。

-----

## 【最重要：出力形式の厳守事項】

### ■ 出力タグ形式（必ず守ること） - 各項目は `<<<タグ名>>>` で開始し、`<<<END>>>` で終了すること - タグ名は完全一致必須（スペース・全角半角の違いでエラーになる） - タグと内容の間に余計な空白・改行を入れないこと - `<<<END>>>` の後は必ず改行すること

### ■ 使用するタグ一覧（これ以外のタグは使用禁止） <<<疾患名>>> <<<発症受傷日>>> <<<合併症>>> <<<既往歴>>> <<<身体機能>>> <<<認知>>> <<<嚥下言語>>> <<<日常生活注意>>> <<<経過>>>

### ■ 「該当なし」の統一表記 - 該当がない場合は必ず「特になし」と記載すること - 以下の表記は禁止：「なし」「該当なし」「特にありません」「記載なし」「ー」「-」

-----

## 【参照と役割（重複防止）】

### ■ 疾患名（リハビリ指示対象疾患） → 電子カルテの「リハビリテーション指示」または「リハビリテーション処方」に記載されている疾患名を抽出する。 → 疾患名のみを記載し、説明や補足は不要。 → 複数ある場合は読点（、）区切りで列挙。 → 例: 右視床出血、左大腿骨頸部骨折

### ■ 発症・受傷日 → 電子カルテ上の「発症日」「受傷日」「手術日」などから主疾患の日付を抽出。 → YYYY/MM/DD 形式で記載（例: 2024/12/15） → 和暦（令和・平成）は禁止。西暦に変換すること。 → 不明な場合は「不明」と記載。

### ■ 合併症 → 電子カルテの「病名登録」から、登録されている病名を全て抽出する。 → 主疾患（リハビリ指示対象疾患）は除外する。 → 疾患名のみを読点（、）区切りで列挙。 → 例: 誤嚥性肺炎、心不全、糖尿病、高血圧、尿路感染症 → 該当なしの場合は「特になし」。

### ■ 既往歴 → 電子カルテの「病名登録」から、登録されている病名を全て抽出する。 → 主疾患（リハビリ指示対象疾患）は除外する。 → 疾患名のみを読点（、）区切りで列挙。 → 例: 脂質異常症、関節リウマチ、骨粗鬆症、前立腺肥大症 → 該当なしの場合は「特になし」。

注意: 合併症と既往歴は電子カルテの病名登録から全ての病名を抽出してください。現在活動性があるか既往かの区別は不要です。登録されている病名を全て列挙してください。

### ■ 身体機能・認知・嚥下言語 → 主にリハ記録（PT/OT/ST）から「現在の状態（評価レベル）」のみを記載する。 → ここでは「改善」「経過」「以前は」などの時系列表現は禁止。

### ■ 日常生活注意 → バイタル記録および病棟ADL記載（看護記録を含む）から、申し送り（行動指示）のみを記載する。 → 原因・評価・経過は禁止。

### ■ 経過 → 入院／転棟／手術／治療／安静度変更／ADLレベル変化など「大きなイベント」＋改善点＋残存課題＋方針のみを記載する。 → 日々の訓練詳細・検査値・点滴・投薬など医療管理情報は禁止。 → 評価名・数値は原則書かない（必要最小限のみ）。

-----

## 【共通ルール（必ず守る）】 1. 推測は禁止。記載がない場合は必ず「特になし」と記載する。 2. 評価名の単純な羅列は禁止。必ず統合して文章化し、ADLへの影響を1フレーズ含める。 3. 出力前に各欄の文字数上限・行数制限を再確認し、必ず上限内に収める。 4. 現在の課題判定は「最新のADL・動作記載」を優先する（評価が再測定されていなくてもよい）。 5. 初期評価で問題があっても、最近のADL記載で問題が示されていなければ現在課題として書かない。 → その場合は経過欄で「改善を認めた」としてのみ使用する。 6. 区切り文字は読点（、）を使用。カンマ（,）や句点（。）での区切りは禁止。

-----

## 【検索対象（表記ゆれ含む）】

### ■ ①疾患名・合併症・既往歴の抽出ルール 疾患名（リハビリ指示）: - 「リハビリテーション指示」「リハ処方」「リハ指示書」欄を優先 - 「主病名」「主疾患」の記載も参照 - 例: 脳梗塞、大腿骨頸部骨折、腰椎圧迫骨折、廃用症候群

合併症（現在治療中）: - 病名登録されている全ての疾患を抽出（主疾患を除く） - 活動性の有無にかかわらず、登録されている病名を全て列挙

既往歴（過去の疾患）: - 病名登録されている全ての疾患を抽出（主疾患を除く） - 活動性の有無にかかわらず、登録されている病名を全て列挙

### ■ ②身体機能（運動・整形・廃用・内部障害・バランス・歩行をすべて含む） - Brunnstrom、ブルンストローム、ブルーストローム、BRS、Brs - SIAS、サイアス、SIAS-M、運動項目、感覚項目、FACT、TIS - MMT、筋力、徒手筋力、筋力低下、握力 - MAS、Modified Ashworth Scale、アシュワース、痙縮、固縮、低緊張 - 疼痛、痛み、NRS、VAS、安静時痛、動作時痛、荷重時痛、階段時痛 - 運動失調、SARA、協調性障害、筋萎縮、サルコペニア

【ROM・可動域の拾い上げルール（超重要）】 ROM という語がなくても、以下は可動域評価として抽出すること。 - 関節名＋運動方向＋数値：例）膝屈曲120、膝関節屈曲120、股関節伸展-5、肩外転90 - 英語混在：knee flex120、hip flex90、shoulder abd80、ankle dorsi10 など - 角度記号がなくても数値があれば可動域として扱う（120、90、-5 等） → これらをROM評価として統合し、ADL制限との関係で要約すること。

### ■ ③バランス・歩行・移動能力（表記ゆれ・同義語辞書） - TUG、Timed Up and Go - 10m歩行、10MWT、歩行速度、m/s、秒 - BBS、Berg、Berg Balance Scale、ベルグ - FBS、Functional Balance Scale、ファンクショナルバランス ※BBSとFBSが混在する場合がある。両方を拾い、正式名称が不明なら「BBS（記載上FBS）」等の注記可。 - Mini-BESTest、mini BEST、ミニベスト、BESTest - SPPB、Short Physical Performance Battery（総合点／サブスコアがあれば抽出） - タンデム立位、タンデム歩行、tandem stance/gait（保持時間や介助要否を抽出） - 片脚立位、片足立ち、OLS、SLS、One Leg Standing - FRT、Functional Reach Test - 転倒歴、ふらつき、よろめき、不安定 - 補助具（杖、歩行器、AFO、装具）

### ■ ④認知・高次脳機能 - MMSE、HDS-R、MoCA、FAB、TMT - 注意障害、遂行機能障害、USN、失行、失認、記憶障害 - 意識変動、せん妄、BPSD、抑うつ、不安、意欲低下

### ■ ⑤嚥下・言語 - 食形態（常食、刻み、ミキサー、ペースト、とろみ） - むせ、誤嚥、湿性嗄声 - VF、VE、RSST、MWST - 失語、構音障害、意思疎通困難、筆談、Yes/No反応

-----

## 【ADL表現ゆれ辞書＋現在課題判定（重要）】

### ▼ 自立レベル（最近の記録でこれがあれば現在課題としない） 自立、見守り不要、単独で可能、介助なし、独歩、歩行自立、移乗自立、トイレ自立、病棟内自立

### ▼ 見守り・軽介助（最近の記録でこれがあれば現在課題として要約してよい） 見守り、声かけ必要、注意必要、軽介助、一部介助、付き添い、不安定、ふらつきあり、疲労時介助増大

### ▼ 明確な介助（最近の記録でこれがあれば必ず現在課題） 中等度介助、全介助、2人介助、支持必要、保持必要、立位保持不可、歩行介助必須

【初期評価のみ存在する項目の扱い】 - 初期評価で問題があっても、最近のADL記載で問題が示されていない場合は現在課題として書かない。 - その場合は経過欄で「改善を認めた」としてのみ使用する。 - 最近のADL記載も無い場合は、推測せず現在課題に書かない（特になし）。

-----

## 【疼痛辞書（現在課題 vs 改善の判定）】

### ▼ 疼痛あり（最近にあれば現在課題） 痛みあり、疼痛訴え、動作時痛、荷重時痛、歩行時痛、立ち上がり時痛、階段時痛、圧痛あり

### ▼ 疼痛なし・軽快（最近にあれば現在課題にしない） 痛み軽減、疼痛軽快、疼痛消失、訴えなし - 初期のみ疼痛があり現在軽快なら、身体機能には書かず経過で改善点として扱う。 - 現在も疼痛が残る場合のみ、身体機能で制限要因として書く。

-----

## 【疲労・耐久性・内部障害辞書（身体機能に集約）】 易疲労、途中休憩必要、距離制限、連続歩行困難、活動量低下、息切れ、呼吸苦、動悸、SpO2低下、酸素使用、心拍数上昇、バイタル変動 - 最近にあれば身体機能の制限要因として要約してよい。 - 改善（休憩不要、息切れなし、SpO2安定、活動量増加、歩行距離延長）が最近にあれば現在課題にしない。 - 呼吸循環・耐久性は必ず身体機能に含め、認知や経過に書かない（経過では生活変化に関係する範囲のみ可）。

-----

## 【ADL場面別リスク辞書（日常生活注意欄専用）】 - 歩行開始時ふらつき／方向転換で不安定／屋外不安定／段差でよろめく／長距離で疲労増大 - 立ち上がり不安定／前方支持必要／移乗時にバランス崩す - トイレ立ち座り不安定／ズボン操作困難／夜間トイレ時転倒リスク - 浴槽出入り困難／更衣時立位不安定 - 階段昇降不安定／手すり必須／下りでふらつき - 疲労で介助量増大／夕方に不安定／連続動作で注意力低下 日常生活注意欄は最大3行、1行 = 1注意点。該当なしは「特になし」。

-----

## 【出力形式（VBA転記用：必ずこの形式で出力）】 重要: 以下の形式を厳密に守ること。タグの前後に余計なスペースや説明を入れないこと。

<<<疾患名>>> 右視床出血、左大腿骨頸部骨折 <<<END>>>

<<<発症受傷日>>> 2024/12/15 <<<END>>>

<<<合併症>>> 誤嚥性肺炎、心不全 <<<END>>>

<<<既往歴>>> 高血圧、糖尿病、脂質異常症 <<<END>>>

<<<身体機能>>> （最大1000字・15行以内） ・現在の障害像（評価レベル）のみ。改善・経過は禁止。 ・数値評価があれば1つ以上は入れる（TUG/10m/BBS/FBS/SPPB/タンデム保持/ROM数値/NRS等）。 ・羅列禁止：統合して文章化し、ADLへの影響を1フレーズ含める。 ・最近のADLが自立レベルなら、初期のみの問題は現在課題にしない。 無ければ「特になし」。 <<<END>>>

<<<認知>>> （最大500字・15行以内） ・現在の状態のみ。改善・経過は禁止。 ・数値があれば使用（MMSE等）。無ければ観察から要約。 ・ADLへの影響は1フレーズで示す。 無ければ「特になし」 <<<END>>>

<<<嚥下言語>>> （最大500字・15行以内） ・現在の安全性のみ。改善・経過は禁止。 ・食形態、むせ、VF/VE、意思疎通の要点。 無ければ「特になし」 <<<END>>>

<<<日常生活注意>>> （最大600字・10行以内） ・申し送り（行動指示）のみ。原因・評価・経過は禁止。 ・場面別辞典から「転倒・事故につながりやすいもの」を優先して記載。 ・1行 = 1注意点が基本。 無ければ「特になし」 <<<END>>>

<<<経過>>> （最大1500字・20行以内） ・大きなイベントのみ（入院、転棟、手術／治療、安静度変更）。 ・改善点：初期（代表）と最新の比較で1～2点（過去のみの課題は書かない）。 ・今後方針・配慮を1文でまとめる。 ・日付は必ず「YYYY年MM月DD日」形式で記載（月だけの記載は禁止） 該当なければ「特になし」

【記載ルール（厳守）】 ・年月または日付が含まれる文は、必ずその文を1行として独立させること。

【記載してもよいイベント（これ以外は書かない）】 ① 入院・転院・発症 ② 転棟・手術・治療開始などの医療上の大きな変化 ③ 退院・転帰・施設入所などの最終決定 ④ 現在の生活レベル／今後方針

【表現テンプレート（必ず準拠）】 YYYY年M月：〇〇のため入院／転院 YYYY年M月：〇〇が可能となった／介助量が軽減した <<<END>>>

-----

## 【出力禁止】 - 氏名、年齢などの基本情報（疾患名・発症日・合併症・既往歴は除く） - FIM/BI点数 - 検査値、点滴、投薬などの医療管理情報 - 評価名の単純羅列 - 同内容の重複記載 - タグの説明や補足（タグと内容のみ出力すること） - 経過欄で「月」のみの記載（必ず「年月日」で記載）

-----

## 【出力例（参考）】

<<<疾患名>>> 右視床出血 <<<END>>>

<<<発症受傷日>>> 2024/12/15 <<<END>>>

<<<合併症>>> 誤嚥性肺炎、心不全 <<<END>>>

<<<既往歴>>> 高血圧、糖尿病、脂質異常症 <<<END>>>

<<<身体機能>>> 右上下肢に中等度の麻痺を認める。BRS上肢III、手指II、下肢IV。10m歩行20秒、TUG25秒。バランスはBBS42点とやや低下している。移乗動作に一部介助を要し、長距離歩行では疲労が増大する。 <<<END>>>

<<<認知>>> MMSE26点。見当識は良好だが、短期記憶にやや低下を認める。日常生活動作の遂行に大きな支障はない。 <<<END>>>

<<<嚥下言語>>> 常食摂取可能。むせや誤嚥なし。意思疎通は良好で、発語明瞭。 <<<END>>>

<<<日常生活注意>>> ・歩行開始時のふらつきに注意 ・夜間トイレ時の転倒リスクあり ・長距離歩行時は休憩を促す <<<END>>>

<<<経過>>> 2024年12月15日：右視床出血のため入院。 2025年1月15日：病棟内歩行が見守りで可能となった。 現在、移乗は一部介助、歩行は見守りレベル。退院後は通所リハビリ継続予定。 <<<END>>>

B. *English Translation*

# Rehabilitation Summary Generation Prompt (Operational Version v4.0)

You should work under the assumption that you can refer to records within the electronic medical record. The purpose is to create a rehabilitation summary that can be understood even by individuals without medical knowledge, including why assistance is needed, what risks exist, how assistance should be provided, and how far the patient has recovered.

-----

## [Most Important: Strict Output Format Requirements]

### ■ Output Tag Format (Must Be Strictly Followed) - Each item must begin with `<<<tag name>>>` and end with `<<<END>>>`. - Tag names must match exactly. Differences in spaces, full-width/half-width characters, or other formatting will cause an error. - Do not insert unnecessary spaces or line breaks between the tag and the content. - Always insert a line break after `<<<END>>>`.

### ■ List of Permitted Tags (Do Not Use Any Tags Other Than These) <<<疾患名>>> <<<発症受傷日>>> <<<合併症>>> <<<既往歴>>> <<<身体機能>>> <<<認知>>> <<<嚥下言語>>> <<<日常生活注意>>> <<<経過>>>

### ■ Standardized Wording for "Not Applicable" - If there is no applicable information, always write 「特になし」. - The following expressions are prohibited: 「なし」, 「該当なし」, 「特にありません」, 「記載なし」, 「ー」, 「-」

-----

## [References and Roles: Preventing Overlap]

### ■ Disease Name (Target Disease for Rehabilitation Order) → Extract the disease name listed in the "rehabilitation order" or "rehabilitation prescription" section of the electronic medical record. → Write only the disease name; no explanation or additional comments are needed. → If there are multiple diseases, list them separated by Japanese commas (、). → Example: right thalamic hemorrhage、left femoral neck fracture

### ■ Date of Onset/Injury → Extract the date of the main disease from the "date of onset," "date of injury," "date of surgery," or similar fields in the electronic medical record. → Use the YYYY/MM/DD format. Example: 2024/12/15 → Do not use Japanese era names such as Reiwa or Heisei. Convert them to the Western calendar. → If unknown, write 「不明」.

### ■ Comorbidities → Extract all registered disease names from the "registered diagnoses" section of the electronic medical record. → Exclude the main disease targeted by the rehabilitation order. → List disease names only, separated by Japanese commas (、). → Example: aspiration pneumonia、heart failure、diabetes mellitus、hypertension、urinary tract infection → If none, write 「特になし」.

### ■ Past Medical History → Extract all registered disease names from the "registered diagnoses" section of the electronic medical record. → Exclude the main disease targeted by the rehabilitation order. → List disease names only, separated by Japanese commas (、). → Example: dyslipidemia、rheumatoid arthritis、osteoporosis、benign prostatic hyperplasia → If none, write 「特になし」.

Note: For both comorbidities and past medical history, extract all disease names from the registered diagnoses in the electronic medical record. It is not necessary to distinguish whether each disease is currently active or part of the past medical history. List all registered disease names.

### ■ Physical Function, Cognition, Swallowing and Language → Primarily use rehabilitation records from PT, OT, and ST, and describe only the current status or current assessment level. → Do not use chronological expressions such as "improved," "course," or "previously" in these sections.

### ■ ADL Precautions → From vital sign records and ward ADL notes, including nursing records, write only handover instructions or action instructions. → Do not include causes, assessments, or clinical course.

### ■ Clinical Course → Write only major events such as admission, ward transfer, surgery, treatment, changes in activity restrictions, changes in ADL level, improvements, remaining problems, and future plans. → Do not include daily training details, laboratory values, intravenous therapy, medications, or other medical management information. → In principle, do not include assessment names or numerical values, except when absolutely necessary.

-----

## [Common Rules: Must Be Followed] 1. Do not infer. If there is no written information, always write 「特になし」. 2. Do not simply list assessment names. Integrate the information into sentences and include one phrase describing the impact on ADL. 3. Before output, recheck the character and line limits for each section and ensure that all sections stay within the limits. 4. When judging current problems, prioritize the most recent ADL and movement-related descriptions, even if assessments have not been repeated. 5. Even if there were problems during the initial assessment, do not describe them as current problems if recent ADL records no longer indicate those problems. → In that case, use them only in the Clinical Course section as "improvement was observed." 6. Use Japanese commas (、) as separators. Do not use English commas (,) or Japanese periods (。) as separators.

-----

## [Search Targets Including Variations in Wording]

### ■ ① Rules for Extracting Disease Name, Comorbidities, and Past Medical History Disease Name (Rehabilitation Order): - Prioritize sections such as "rehabilitation order," "rehabilitation prescription," and "rehabilitation instruction form." - Also refer to entries such as "main diagnosis" or "main disease." - Examples: cerebral infarction, femoral neck fracture, lumbar compression fracture, disuse syndrome

Comorbidities (Currently Being Treated): - Extract all registered diseases, excluding the main disease. - List all disease names registered in the medical record, regardless of whether they are active.

Past Medical History (Past Diseases): - Extract all registered diseases, excluding the main disease. - List all disease names registered in the medical record, regardless of whether they are active.

### ■ ② Physical Function (Includes Motor Function, Orthopedic Conditions, Disuse, Internal Disorders, Balance, and Gait) - Brunnstrom, Brunnstrom stage, BRS, Brs - SIAS, SIAS-M, motor items, sensory items, FACT, TIS - MMT, muscle strength, manual muscle testing, muscle weakness, grip strength - MAS, Modified Ashworth Scale, Ashworth, spasticity, rigidity, hypotonia - Pain, NRS, VAS, resting pain, movement-related pain, weight-bearing pain, stair-related pain - Ataxia, SARA, coordination disorder, muscle atrophy, sarcopenia

[Rules for Identifying ROM and Range of Motion: Extremely Important] Even if the term ROM is not used, the following should be extracted as range-of-motion assessments: - Joint name + movement direction + numerical value. Examples: knee flexion 120, knee joint flexion 120, hip extension -5, shoulder abduction 90 - Mixed English notation: knee flex120, hip flex90, shoulder abd80, ankle dorsi10, etc. - If a numerical value is present, treat it as range-of-motion information even if the degree symbol is not written (e.g., 120, 90, -5). → Integrate these as ROM assessments and summarize them in relation to ADL limitations.

### ■ ③ Balance, Gait, and Mobility (Dictionary of Wording Variations and Synonyms) - TUG, Timed Up and Go - 10-meter walk test, 10m walking, 10MWT, walking speed, m/s, seconds - BBS, Berg, Berg Balance Scale - FBS, Functional Balance Scale Note: BBS and FBS may be mixed in the record. Extract both. If the formal name is unclear, a note such as "BBS (written as FBS in the record)" may be used. - Mini-BESTest, mini BEST, BESTest - SPPB, Short Physical Performance Battery (extract total and sub-scores if available) - Tandem standing, tandem gait, tandem stance/gait (extract holding time and need for assistance) - Single-leg standing, one-leg standing, OLS, SLS, One Leg Standing - FRT, Functional Reach Test - Fall history, unsteadiness, staggering, instability - Assistive devices such as a cane, walker, AFO, orthosis

### ■ ④ Cognition and Higher Brain Function - MMSE, HDS-R, MoCA, FAB, TMT - Attention disorder, executive dysfunction, unilateral spatial neglect, apraxia, agnosia, memory impairment - Fluctuating consciousness, delirium, BPSD, depression, anxiety, reduced motivation

### ■ ⑤ Swallowing and Language - Diet form: regular diet, chopped diet, mixer diet, paste diet, thickened liquids - Choking, aspiration, wet hoarseness - VF, VE, RSST, MWST - Aphasia, dysarthria, difficulty communicating, writing communication, yes/no response

-----

## [ADL Wording Variation Dictionary and Current Problem Judgment: Important]

### ▼ Independent Level (If present in recent records, do not treat as a current problem) Independent, no supervision required, able to perform alone, no assistance, independent walking, walking independent, transfer independent, toilet independent, independent within the ward

### ▼ Supervision or Minimal Assistance (If present in recent records, may be summarized as a current problem) Supervision, verbal cues required, caution required, minimal assistance, partial assistance, accompaniment, unstable, unsteadiness present, increased assistance when fatigued

### ▼ Clear Assistance (If present in recent records, must be treated as a current problem) Moderate assistance, total assistance, two-person assistance, support required, holding required, unable to maintain standing, walking assistance essential

[Handling Items Present Only in the Initial Assessment] - Even if a problem was present in the initial assessment, do not write it as a current problem if recent ADL records do not indicate the problem. - In that case, use it only in the Clinical Course section as "improvement was observed." - If there are no recent ADL records either, do not infer and do not write it as a current problem (write 「特になし」).

-----

## [Pain Dictionary: Judgment of Current Problem vs Improvement]

### ▼ Pain Present (If present recently, treat as a current problem) Pain present, complaint of pain, movement-related pain, weight-bearing pain, pain during walking, pain during standing up, stair-related pain, tenderness present

### ▼ No Pain or Improved Pain (If present recently, do not treat as a current problem) Pain decreased, pain improved, pain disappeared, no complaint - If pain was present only initially and has now improved, do not write it in Physical Function; treat it as an improvement in the Clinical Course section. - Only when pain still remains at present should it be written in Physical Function as a limiting factor.

-----

## [Fatigue, Endurance, and Internal Disorder Dictionary: Integrated Into Physical Function] Easy fatigability, need for rest breaks, distance limitation, difficulty with continuous walking, reduced activity level, shortness of breath, respiratory distress, palpitations, decreased SpO_2_, oxygen use, increased heart rate, vital sign fluctuation - If present recently, these may be summarized as limiting factors in Physical Function. - If recent records indicate improvement (no need for rest, no shortness of breath, stable SpO_2_, increased activity level, longer walking distance), do not treat them as current problems. - Respiratory, circulatory, and endurance-related issues must always be included in Physical Function and should not be written in Cognition or Clinical Course (in Clinical Course, only the extent related to changes in daily life is permitted).

-----

## [ADL Situation-Specific Risk Dictionary: For the ADL Precautions Section Only] - Unsteadiness when starting to walk/instability when changing direction / outdoor instability / staggering on steps / increased fatigue with long-distance walking - Unstable sit-to-stand/need for anterior support/loss of balance during transfer - Instability when sitting down on or standing up from the toilet/difficulty managing pants/fall risk during nighttime toileting - Difficulty entering or exiting the bathtub/standing instability during dressing - Unstable stair climbing/handrail required/unsteadiness when descending stairs - Increased assistance due to fatigue/instability in the evening/reduced attention during continuous activity The ADL Precautions section should be a maximum of three lines, with one precaution per line. If none, write 「特になし」.

-----

## [Output Format for VBA Transcription: Strictly Follow This Format] Important: Strictly follow the format below. Do not add unnecessary spaces or explanations before or after the tags.

<<<疾患名>>> 右視床出血、左大腿骨頸部骨折 <<<END>>>

<<<発症受傷日>>> 2024/12/15 <<<END>>>

<<<合併症>>> 誤嚥性肺炎、心不全 <<<END>>>

<<<既往歴>>> 高血圧、糖尿病、脂質異常症 <<<END>>>

<<<身体機能>>> (Maximum 1,000 Japanese characters and within 15 lines) ・Only the current impairment status or current assessment level should be written. Do not include improvement or clinical course. ・If numerical assessments are available, include at least one value (e.g., TUG, 10-meter walk, BBS/FBS, SPPB, tandem holding time, ROM value, NRS). ・Do not simply list assessments. Integrate the information into sentences and include one phrase describing the impact on ADL. ・If recent ADL is at an independent level, do not treat problems present only initially as current problems. If none, write 「特になし」. <<<END>>>

<<<認知>>> (Maximum 500 Japanese characters and within 15 lines) ・Only the current status should be written. Do not include improvement or clinical course. ・Use numerical values if available (e.g., MMSE). If not available, summarize based on observation. ・Describe the impact on ADL in one phrase. If none, write 「特になし」. <<<END>>>

<<<嚥下言語>>> (Maximum 500 Japanese characters and within 15 lines) ・Only current safety should be written. Do not include improvement or clinical course. ・Summarize key points regarding diet form, choking, VF/VE, and communication. If none, write 「特になし」. <<<END>>>

<<<日常生活注意>>> (Maximum 600 Japanese characters and within 10 lines) ・Write only handover or action instructions. Do not include causes, assessments, or clinical course. ・Prioritize items from the situation-specific dictionary that are likely to lead to falls or accidents. ・As a rule, one line should contain one precaution. If none, write 「特になし」. <<<END>>>

<<<経過>>> (Maximum 1,500 Japanese characters and within 20 lines) ・Write only major events such as admission, ward transfer, surgery or treatment, and changes in activity restrictions. ・Improvement points: compare the representative initial status and the latest status in one or two points (do not write problems that existed only in the past). ・Summarize future plans and considerations in one sentence. ・Any date must be written in the YYYY年MM月DD日 format (month-only entries are prohibited). If none, write 「特になし」.

[Writing Rules: Strictly Follow] ・Any sentence that includes a year/month or date must be written as an independent line.

[Permitted Events to Write: Do Not Write Anything Other Than These] ① Admission, transfer from another hospital, or onset ② Major medical changes such as ward transfer, surgery, or the start of treatment ③ Final decisions such as discharge, outcome, or facility admission ④ Current living level and future plan

[Expression Templates: Must Follow] YYYY年M月: admitted/transferred due to 〇〇 YYYY年M月: became able to 〇〇/amount of assistance decreased <<<END>>>

-----

## [Prohibited Output] - Basic information such as name and age, except for disease name, date of onset, comorbidities, and past medical history - FIM or BI scores - Laboratory values, intravenous therapy, medications, and other medical management information - Simple lists of assessment names - Duplicate descriptions of the same content - Explanations or supplementary comments about tags; output only tags and content - Month-only date entries in the Clinical Course section (dates must always be written as year/month/day)

-----

## [Output Example: Reference]

<<<疾患名>>> 右視床出血 <<<END>>>

<<<発症受傷日>>> 2024/12/15 <<<END>>>

<<<合併症>>> 誤嚥性肺炎、心不全 <<<END>>>

<<<既往歴>>> 高血圧、糖尿病、脂質異常症 <<<END>>>

<<<身体機能>>> The patient has moderate paralysis of the right upper and lower limbs. BRS: upper limb III, fingers II, lower limb IV. 10-meter walk: 20 seconds; TUG: 25 seconds. Balance is mildly impaired, with a BBS score of 42. Partial assistance is required for transfers, and fatigue increases during long-distance walking. <<<END>>>

<<<認知>>> MMSE score is 26. Orientation is good, but mild short-term memory impairment is present. There is no major interference with performance of activities of daily living. <<<END>>>

<<<嚥下言語>>> Regular diet is possible. No choking or aspiration is observed. Communication is good, and speech is clear. <<<END>>>

<<<日常生活注意>>> ・Be careful of unsteadiness when starting to walk. ・There is a fall risk during nighttime toileting. ・Encourage rest breaks during long-distance walking. <<<END>>>

<<<経過>>> 2024年12月15日: admitted due to right thalamic hemorrhage. 2025年1月15日: became able to walk within the ward under supervision. Currently, transfers require partial assistance and walking is at the supervision level. Continued outpatient/day-care rehabilitation is planned after discharge. <<<END>>>
