# Correction: Implementation of an AI-Assisted Workflow for Rehabilitation Discharge Summary Creation: Evaluation of Documentation Efficiency, Usability, and Hallucinations in a 290-Bed General Hospital in Japan

**DOI:** 10.7759/cureus.c496

**Published:** 2026-10-30

**Authors:** Masaji Nakano, Takuto Imura

**Affiliations:** 1 Rehabilitation, Soseikai General Hospital, Kyoto, JPN

This article has been corrected to replace Figure 1 and to correct minor errors in the Abstract and Results sections. These changes do not affect the results or conclusions of the article.

